# Fetal Behavioural Responses to Maternal Voice and Touch

**DOI:** 10.1371/journal.pone.0129118

**Published:** 2015-06-08

**Authors:** Viola Marx, Emese Nagy

**Affiliations:** School of Psychology, University of Dundee, Dundee, United Kingdom; Università di Parma, ITALY

## Abstract

**Background:**

Although there is data on the spontaneous behavioural repertoire of the fetus, studies on their behavioural responses to external stimulation are scarce.

**Aim, Methods:**

The aim of the current study was to measure fetal behavioural responses in reaction to maternal voice; to maternal touch of the abdomen compared to a control condition, utilizing 3D real-time (4D) sonography. Behavioural responses of 23 fetuses (21st to 33rd week of gestation; N = 10 in the 2nd and N = 13 in the 3rd trimester) were frame-by-frame coded and analyzed in the three conditions.

**Results:**

Results showed that fetuses displayed more arm, head, and mouth movements when the mother touched her abdomen and decreased their arm and head movements to maternal voice. Fetuses in the 3rd trimester showed increased regulatory (yawning), resting (arms crossed) and self-touch (hands touching the body) responses to the stimuli when compared to fetuses in the 2nd trimester.

**Conclusion:**

In summary, the results from this study suggest that fetuses selectively respond to external stimulation earlier than previously reported, fetuses actively regulated their behaviours as a response to the external stimulation, and that fetal maturation affected the emergence of these differential responses to the environment.

## Introduction

The mother was once regarded as a vehicle, a conduit for nutrition and waste removal for the fetus that lived isolated from the outside world [1–4]. Recent research using ultrasound techniques however, started to accumulate evidence on the impact of the external world on the fetus [2–8].

Newborns preferentially respond to maternal voice hours after birth [9–13], suggesting that the fetus is able to detect stimuli in utero and form memories of them. The earliest fetal responses to sound were reported at 16 weeks of gestation [14], much before the development of the fetal ear is complete.

Previous studies on fetal responses to maternal voice measured changes in fetal heart rate (FHR) and lead to inconclusive results. Kisilevsky et al. [15–16] found an increase, whereas [17] and [18] reported a decrease in FHR in response to maternal voice. An increase in FHR might indicate an arousal response, whereas a decrease may suggest a possible orientating mechanism to maternal voice [11]. Hepper, Scott, & Shahidullah [19] however, found that the voice of the mother affected FHR the same way as did the voice of a female stranger with no differential FHR.

A possible reason for the varying outcomes of these studies might be due to methodological differences. It is likely that presenting the maternal voice indirectly such as recorded voice and via headphones, rather than through bone and fluid conduction within the body as it happens with natural speech, may result in an altered sound experience for the fetus. Indeed, when Hepper et al. [19] administered maternal voice both ‘in situ’ and pre-recorded, they found that fetuses at 36 weeks of gestational age (GA) increased FHR responses to the recording but not to natural maternal voice.

Proprioception, sensitivity to touch, develops from 8 weeks GA and by 32 weeks GA most of the body is sensitive to the light stroke of a feather [20]. Previous research reported increases in FHR to vibration from 26 weeks GA with stable and consistent FHR increase by 32 weeks GA [21]. It was also reported that in early pregnancy fetuses tend to move away from stimuli that touch their bodies, whereas later on they tend to move towards them [22].

In summary, previous studies show a) inconclusive results on FHR to maternal voice and b) that although the fetus is sensitive to proprioceptive stimulation and maternal touch on the abdomen is a very commonly occurring natural stimulus for the fetus, there is currently no research which has investigated the effect of maternal touch of the abdomen on fetal responses. Additionally, although there are reports on the spontaneous behavioural repertoire of the fetus, such as fine and gross motor movements, facial expressions, self-touch and yawning [23–25], studies on behavioural responses to external stimulation are scarce.

The aim of the current study was to measure fetal behavioural responses in reaction to maternal voice in situ and to maternal touch of the abdomen as well as in control, no sound, no touch, utilizing 3D real-time (4D) sonography. Based on previous research [18–19], it is hypothesised that fetuses will exhibit a similar attentional orientation-response to that of the newborn [26]. Although no prior research on fetal behavioural responses to maternal touch exists, it is expected that fetuses, in particular older fetuses [22] will respond to touch with a selective increase in movement, when compared to a control condition with no stimulation or to maternal voice.

## Materials and Methods

The study has been reviewed and approved by the Ethical Committee of the University of Dundee (Approval Number: DREC14003). Written consent and permission was also obtained to use the images presented in this article for illustration.

### Participants

23 low-risk expecting mothers (aged 18–35 years, mean = 27.82 years, *SD* = 3.97) who signed the informed consent were included in the study. All mothers had singleton pregnancies and were between the 21^st^ to 33^rd^ week of gestation (Mean = 27.09 weeks, *SD* = 4.07). Included mothers 1) did not smoke, drink or use drugs during pregnancy, 2) had no health and obstetrical complications during pregnancy (including normal blood pressure, amniotic fluid), and 3) had their 20 week check-up scan in order to ensure the health of the fetus prior to participation. All mothers were English native speakers. Twelve mothers were primiparas, 11 mothers had one or more children (mean = 1.22 children, *SD* = 1.88). Eleven mothers were in long-term relationship, 7 were married, 2 engaged, 1 single and 2 were divorced. All mothers were interviewed prior participation regarding any recent environmental stressor, and none reported any major stressors.

### Procedure

The experiment took place in the morning in a semi-darkened room of the Developmental Neuropsychology laboratory of the School of Psychology at the University of Dundee. Mothers lay on a scanning bed, with a pillow behind their heads. The experimenter sat next to the participant with the ultrasound system 'GE Voluson i' with a 'RAB4-8-RS4D' probe, set to 4 frames per second capturing the upper torso including the face of the fetus. A 27-inch monitor was positioned at the end of the bed so that the participant was able to follow the scan.

Each mother participated in three conditions. The ‘Voice’ condition required the participant to read either of two stories (Little Three Pigs [27] or Jack and the Beanstalk [28]) counterbalanced across all participants. In the ‘Touch’ condition mothers touched their abdomen as they usually would—stroking and rubbing the abdomen without interfering with the ultrasound probe and in the ‘Control’ condition mothers lay quietly with hands beside their bodies.

Each block began with a baseline period followed by the stimulus (touch, voice, control, depending on condition), followed by a second baseline period. Each section lasted for 3 minutes, thus each session lasted for 9 minutes, resulting in a total scanning time of 27 minutes per participant. The order of the three conditions was randomized and counterbalanced across all participants.

### Ultrasound recording

A 'GE Voluson i' Ultrasound System with a 'RAB4-8-RS4D' probe as well as ultrasound gel was used to perform the 3D/4D scans. The entire scan was recorded on a 'MacBook Pro’ via an 'Elgato Game Capture HD', High Definition Game Recorder, which was connected via a VGA to HDMI converter to the ultrasound system itself. Furthermore an HDMI signal was outputted via the 'Elgato Game Capture HD' to a 27-inch television so that participants could follow the scan. The incoming signal was recorded using 'Game Capture HD' software for 'MAC OS X' from Elgato. A 'Sony HDR CX220E ' on a tripod was used to record both video and audio for the session, framing the participant’s face and stomach as well as part of the ultrasound system in order to synchronise recordings for later analyses.

Fetal wakefulness was assessed using ultrasound prior to the start of the experiment and the experiment started when the fetus was awake. The fetus was visualised using 4-D colour ultrasound as well as sequences of traditional 2D ultrasound when 4D acquisition was not possible.

### Coding

Movements of arms, head, mouth, arms crossing, hands touching the body and yawning were coded. The first minute of each condition was coded for each participant, frame-by-frame, using the Noldus Observer-Pro 5.0 system (Noldus Information Technology, 2009). The coder was blind to the actual condition and the identity of the fetus.

**Fig 1:**
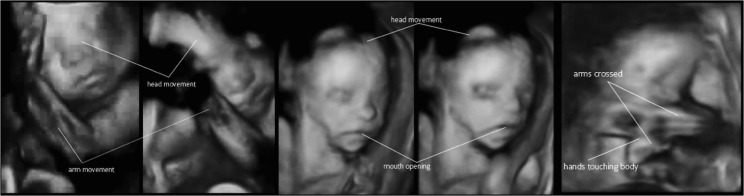
4D illustrations of fetuses displaying arm, head, and mouth movements; the hands touching the body and the arms in crossed position.

‘Arm movements’ were coded when fetuses moved their arms, up or down or displayed arm rotation. ‘Self-touch’ was coded when fetuses touched their own body, own face or the uterus wall. “Arms crossed” was coded when the fetus crossed the arms. ‘Mouth movements’ were opening and closing the mouth. Yawning was coded when the fetus visibly yawned. All behaviours were coded for their frequencies, frequencies being defined as the number of movements per minute. (See Fig 1 for illustration).

#### Reliability coding

13% of the data from each condition were reliability coded by a trained second coder. Inter-rater reliabilities ranged from 64% to 86% with an average of 78% and Cohen’s kappas ranged from 0.61 to 0.85 with an average of .77.

#### Statistical analysis

Frequencies of the behaviours (rate/minute) were calculated using the Observer XT-9.0 system (Noldus Information Technology, 2009) and then subjected to further statistical analysis. Repeated Analysis of Variances (ANOVAs) were conducted using SPSS 19.0 for Windows statistical software (SPSS, Inc., Chicago, IL), and a *p* < .05 was accepted as significant throughout. When Mauchley’s test indicated a violation of the assumption of sphericity, degrees of freedom were corrected using Greenhouse-Geisser sphericity estimates.

## Results

3 x 2 mixed design ANOVAs were conducted on the effect of the three conditions (‘Condition’: Control, Voice, Touch) and the gestational age of the fetus (‘GA’: second (< 26 weeks, *n* = 10) and third trimester (> = 26 weeks, *n* = 13)) on the frequencies of the frequencies of arm movements, arm-cross, head movements, mouth movements, hand-body touch and yawning behaviours.

When Mauchly’s test of sphericity was significant, either the Greenhouse-Geisser or Huynh-Feldt correction was used, depending on relevant epsilon values. Moreover, when Levene’s test of equality of error variances was significant an independent samples Mann-Whitney U test was calculated. All pairwise post-hoc comparisons were conducted using Bonferroni corrections.

### Arm movements

**Fig 2:**
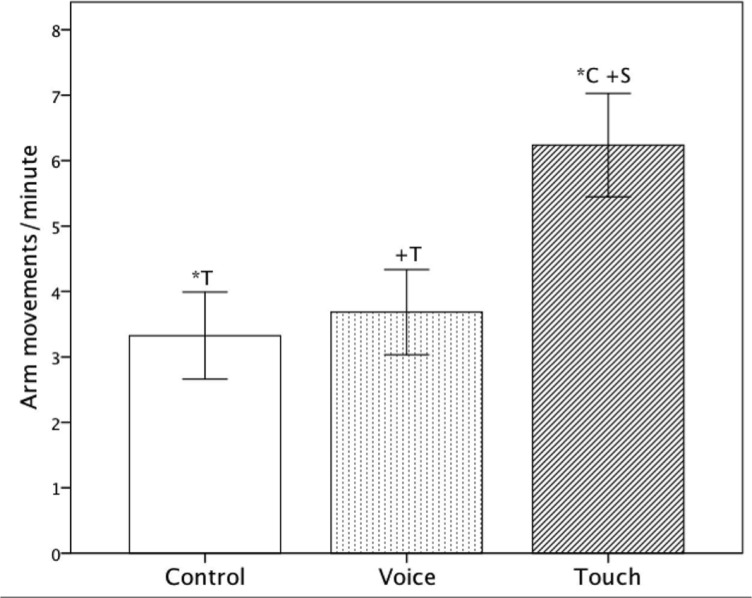
Average frequency of arm movements per minute including standard errors for all three conditions (‘Control’, ‘Voice’ and ‘Touch’). +: p<.10 *: p<.05

There was a significant main effect of ‘Condition’ on arm movements, *F*(2, 42) = 5.84, *p* = .006, η_p_^2^ = .22, however there was no significant main effect of ‘GA’ (Mann-Whitney U: *p* = .580). No significant interaction between ‘Condition’ and ‘GA’ (*F*(2, 42) = 1.26, *p* = .30) was found. Post-hoc pairwise comparison indicated that fetuses displayed significantly more arm movements in ‘Touch’ as compared to ‘Control’ (‘Touch’: Mean = 6.26, *SE* = .80, ‘Control’: Mean = 3.28, *SE* = .65, *p* = .014), as well as a non-significant trend indicating fewer arm movements in ‘Voice’ (Mean = 3.65, *SE* = .64, *p* = .074) as compared to ‘Touch’. ‘Control’ and ‘Voice’ were not statistically different (Table 1 and Fig 2.).

**Table 1:** Results from the mixed ANOVAs for the five coded movements.

Condition		Control	Voice	Touch	Main effect: Condition	Main effect: GA	Condition*GA
Arm movements	Mean	3.28	3.65	6.26	F (2, 42) = 5.84, p = .006, η_p_^2^ = .22	Mann-Whitney U p = .580	F (2, 42) = 1.26, p = .30
	SE	.65	.64	.80			
	Control		NS	.014			
	Voice	NS		.074			
	Touch	.014	.074				
Arms-crossed	Mean	.30	.37	.00	F (2, 42) = 6.66, p = .003, η_p_^2^ = .24	Mann-Whitney U p = .039	F (2, 42) = 6.27, p = .004
	SE	.12	.11	.00			
	Control		NS	.054			
	Voice	NS		.01			
	Touch	054	.01				
Head movements	Mean	2.78	2.56	5.32	F (2, 42) = 4.17, p = .022, η_p_^2^ = .17	F (1, 21) = .30, p = .591	F (2, 42) = .54, p = .584
	SE	.79	.73	.94			
	Control		NS	NS			
	Voice	NS		.061			
	Touch	NS	.061				
Mouth Movements	Mean	.34	.55	1.27	F (2, 42) = 3.45, p = .041, η_p_^2^ = .14,	Mann-Whitney U p = 1.00	F (2, 42) = 1.28, p = .288
	SE	.13	.23	.41			
	Control		NS	.077			
	Voice	NS		NS			
	Touch	.077	NS				
Yawning	Mean	.16	.04	.09	F (2, 42) = 1.09, p = .344	Mann-Whitney U p = .449	F (2, 42) = 3.71, p = .033
	SE	.07	.04	.06			
	Control		NS	NS			
	Voice	NS		NS			
	Touch	NS	NS				

### Hands touching the body

While ‘Condition’ had no effect on the frequency of this behaviour, *F*(2, 42) = .19, *p* = .787, Mann-Whitney U revealed a significant main effect of ‘GA’ (*p* = .033). The ‘Condition’*’GA’ interaction however, was not significant (*F*(2, 42) = .46, *p* = .597).

**Fig 3:**
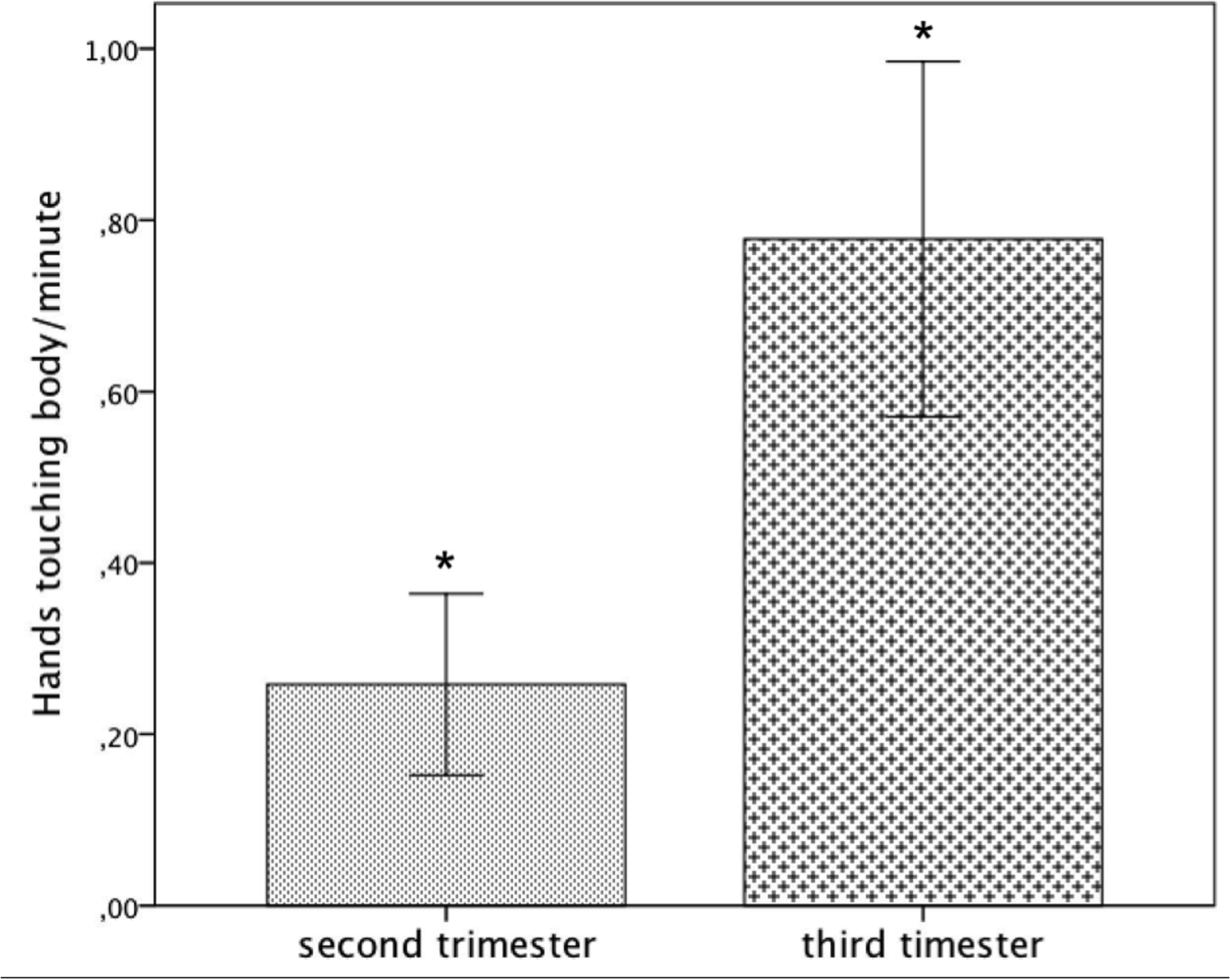
Average frequency of hands touching the body per minute including standard errors for GA (second and third trimester). *: p<.05.

Post-hoc pairwise comparison showed that the frequency of hands touching the body was significantly higher in the third trimester as compared to the second trimester (second trimester: Mean = .26, *SE* = .15, third trimester: Mean = .74, *SE* = .14, *p* = .028). This suggests that older fetuses displayed significantly more body contact in contrast to younger fetuses (Fig 3).

### Arms Crossing

There was a significant main effect of ‘Condition,’ (*F*(2, 42) = 6.66, *p* = .003, η_p_^2^ = .24), a significant main effect of ‘GA’ (Mann-Whitney U: *p* = .039) and a significant ‘Condition’ and ‘GA’ interaction (*F*(2, 42) = 6.27, *p* = .004).

**Fig 4:**
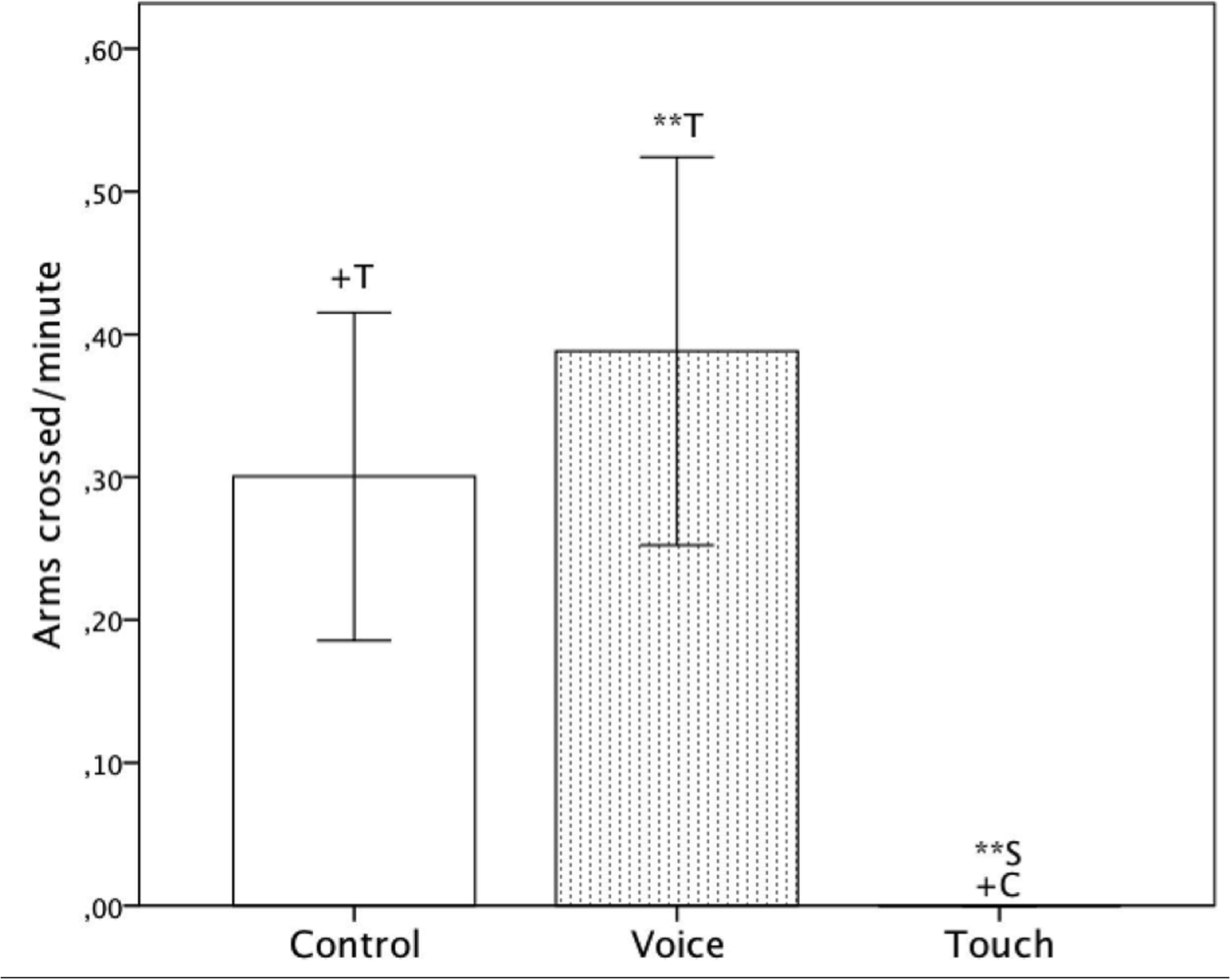
Average frequency of arms crossed per minute including standard errors for all three conditions (‘Control’, ‘Voice’ and ‘Touch’). +: p<.10 **: p<.01.

Post-hoc pairwise comparison showed that the frequency of the arms being crossed was significantly lower ‘Touch’ compared to ‘Voice’ (‘Voice’: Mean = .37, *SE* = .11, ‘Touch’: Mean = .00, *SE* = .00, *p* = .01) and non-significantly lower than in ‘Control’ (‘Control’: Mean = .30, *SE* = .12, *p* = .054) and ‘Touch’ as shown in Fig 4 and on Table 1. The frequency of the arms cross during ‘Control’ and ‘Voice’ were not statistically different.

**Fig 5:**
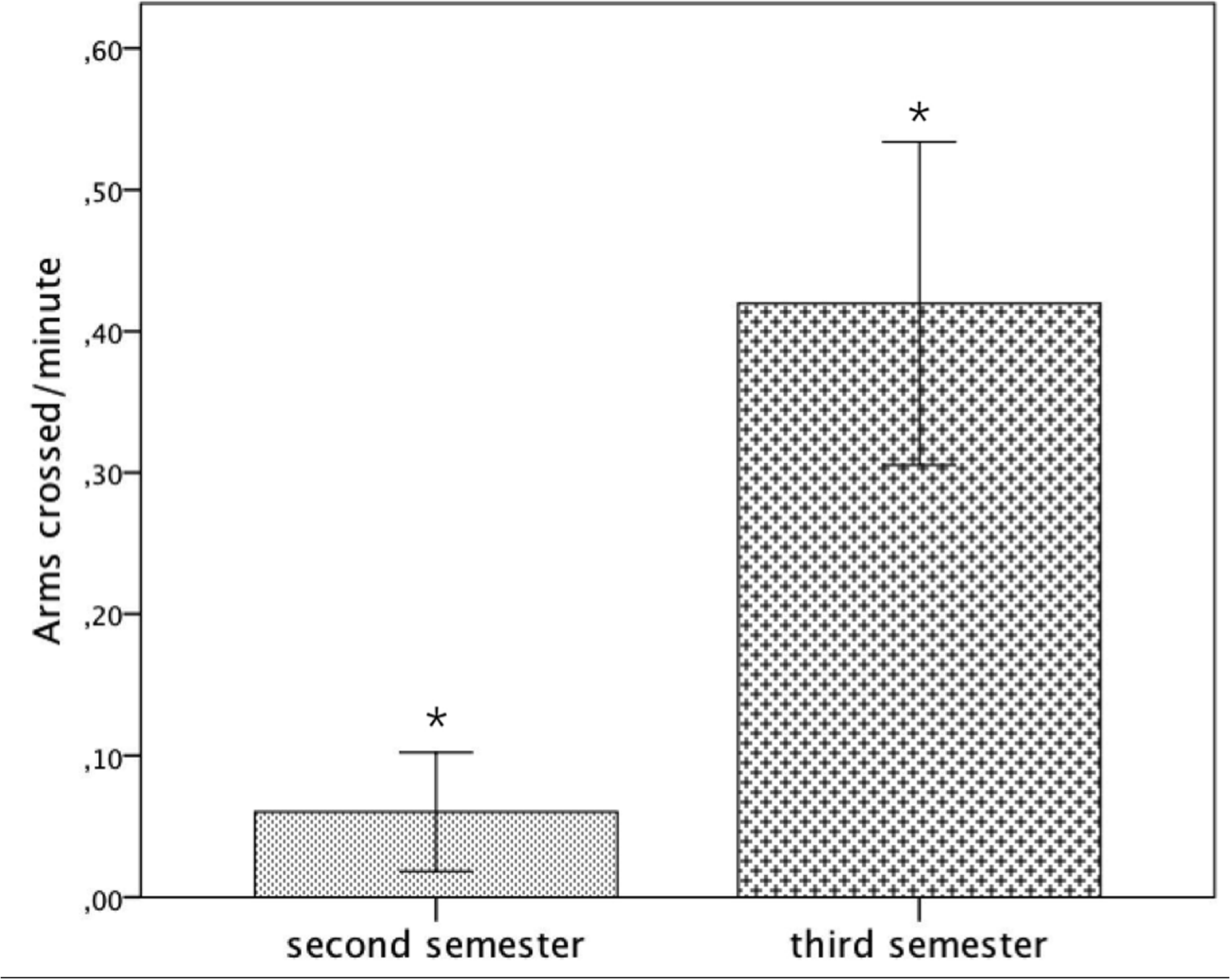
Average frequency of arms crossed per minute including standard errors for GA (second and third trimester). *: p<.05.

Fetuses in the third trimester showed more arm-cross compared to fetuses in the second trimester (third trimester: Mean = .39, *SE* = .09, second trimester: Mean = .06, *SE* = .10, *p* = .029) (Fig 5).

Older fetuses in the third trimester showed no arm-cross movement in the ‘Touch’ condition and that was significantly lower than in the ‘Voice’ condition (‘Touch’: Mean = .00, *SE* = .00, ‘Voice’: Mean = .74, *SE* = .16, *p* < .001) and marginally lower than in the ‘Control’ condition (‘Control’: Mean = .41, *SE* = .16, *p* = .054). The arm cross movements were most frequent in the ‘Voice’ condition, marginally higher than in the ‘Control’ (*p* = .052).

**Fig 6:**
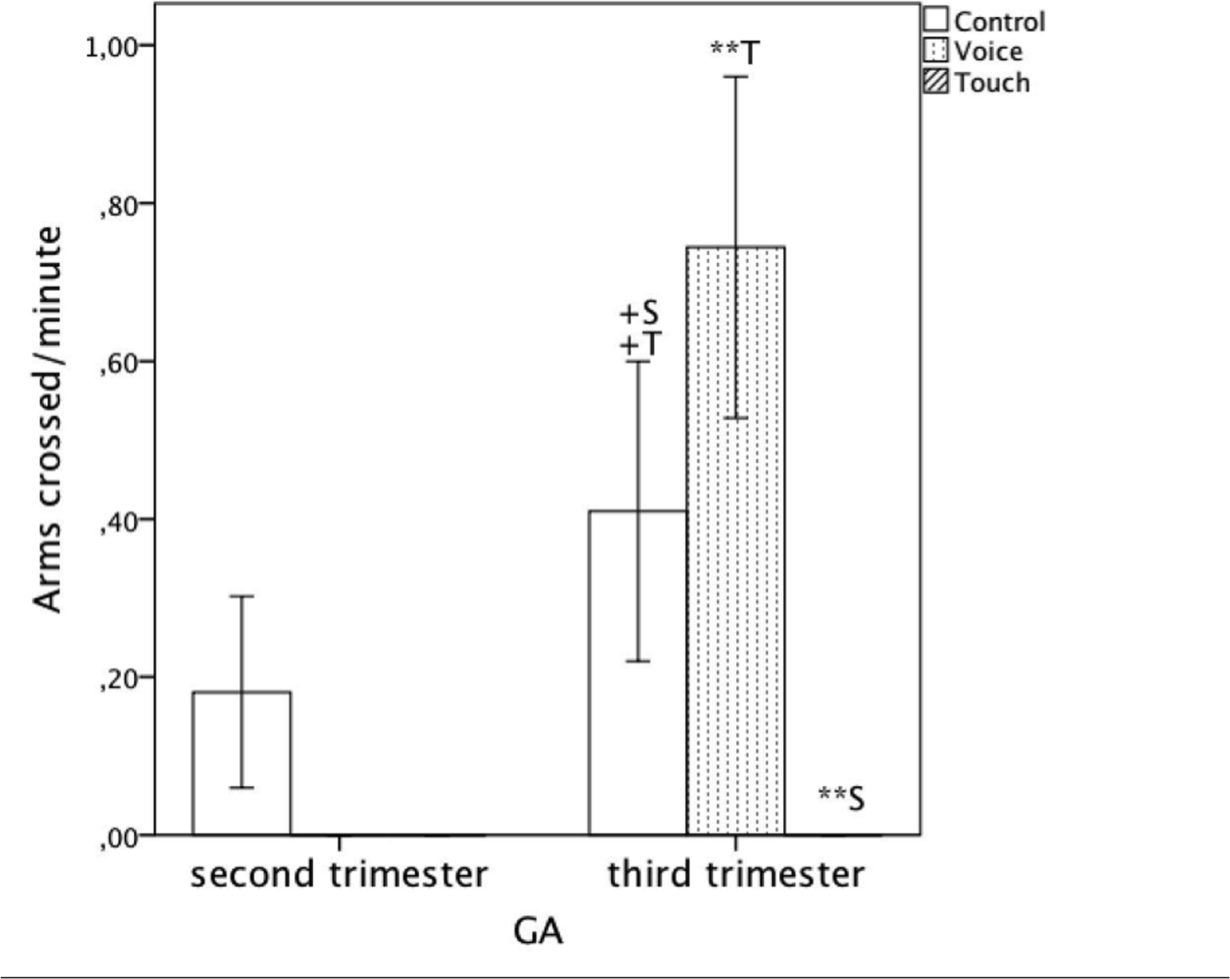
Average frequency of arms crossed per minute including standard errors for all three conditions (‘Control’, ‘Voice’ and ‘Touch’) across second and third trimester. +: p<.10. **: p<.01.

Younger fetuses however, showed no difference in frequencies of arm cross movements across the three conditions (See Fig 6 and Table 2).

**Table 2:** Means, Standard Deviations and significance of pairwise comparisons for conditions and ‘GA’ on the frequency of arms crossed movements of the fetus.

	Second trimester (<26 weeks)			Third trimester (> = 26 weeks)		
Conditions	Control	Voice	Touch	Control	Voice	Touch
Mean	.18	-1.11E-16	.00	.41	.74	.00
SE	.17	.16	.00	.16	.16	.00
Control		N.S.	N.S.		p = .052	p = .053
Voice	N.S.		N.S.	p = .052		p< .001
Touch	N.S.	N.S.		p = .053	p< .001	

### Head movements

**Fig 7:**
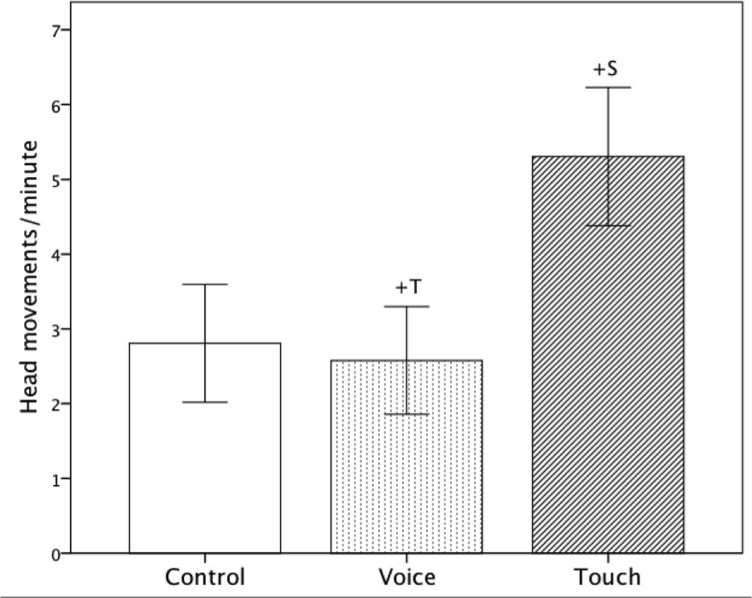
Average frequency of head movements per minute including standard errors for all three conditions (‘Control’, ‘Voice’ and ‘Touch’). +: p<.10.

There was a significant main effect of ‘Condition,’ (*F*(2, 42) = 4.17, *p* = .022, η_p_^2^ = .17), that fetuses showed marginally more head movements in the ‘Touch’ than in the ‘Voice’ conditions (‘Touch’: Mean = 5.32, *SE* = .94, ‘Voice’: Mean = 2.56, *SE* = .73, *p* = .061). No further effects were observed (Fig 7 and Table 1).

### Mouth movements

**Fig 8:**
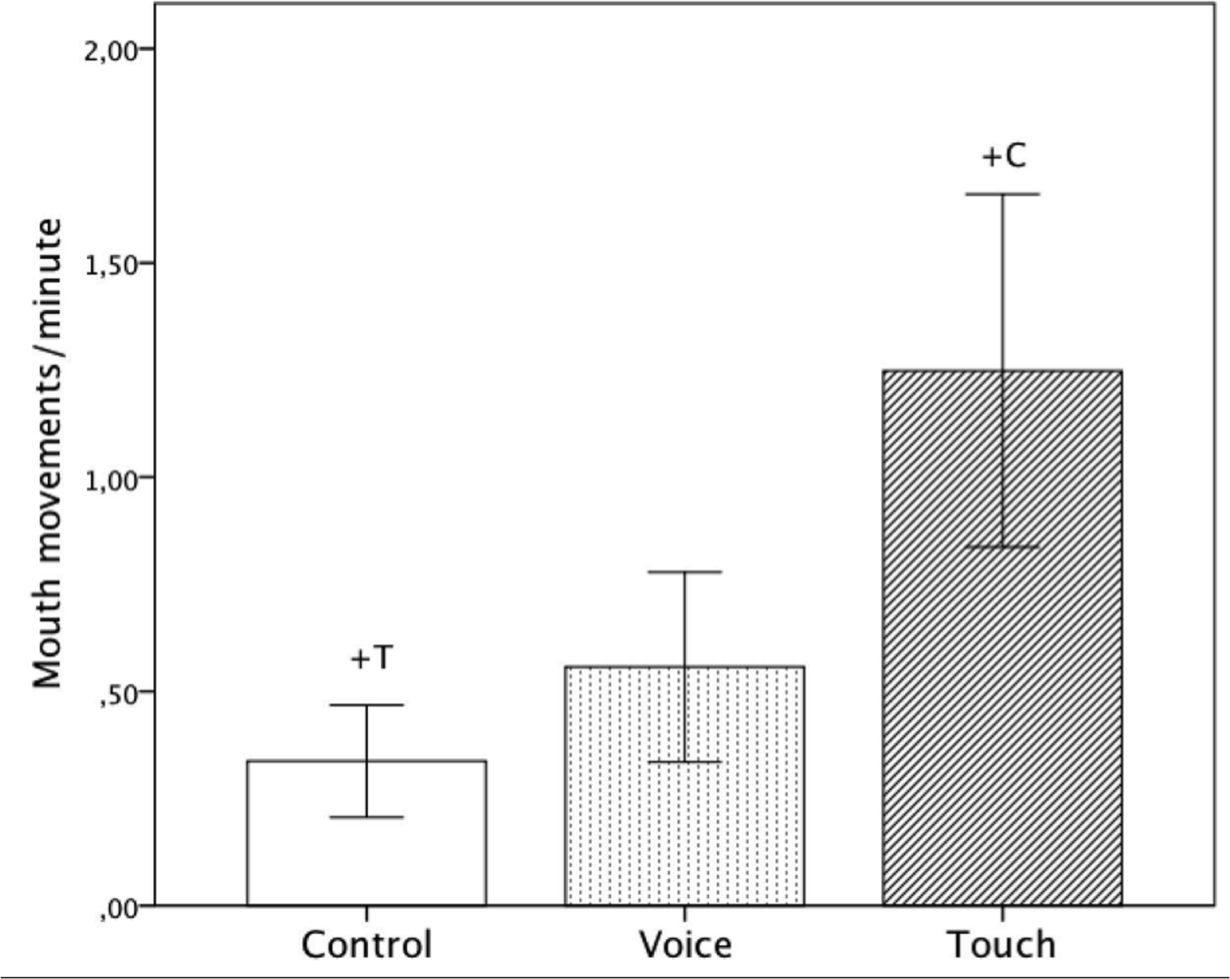
Average frequency of mouth movements per minute including standard errors for all three conditions (‘Control’, ‘Voice’ and ‘Touch’). +: p<.10.

There was a significant main effect of ‘Condition’ (*F*(2, 42) = 3.45, *p* = .041, η_p_^2^ = .14.) The frequency of mouth movements were greater in the ‘Touch’ compared to the ‘Control’ condition (‘Touch’: Mean = 1.27, *SE* = .41, ‘Control’: Mean = .34, *SE* = .23, *p* = .077), although the pairwise comparison did not reach the level of significance. (Fig 8 and Table 1) No further effects were observed.

### Yawning

There was a significant interaction between ‘Condition’ and ‘GA’ (*F*(2, 42) = 3.71, *p* = .033, η_p_^2^ = .15) on the frequency of the yawning.

**Fig 9:**
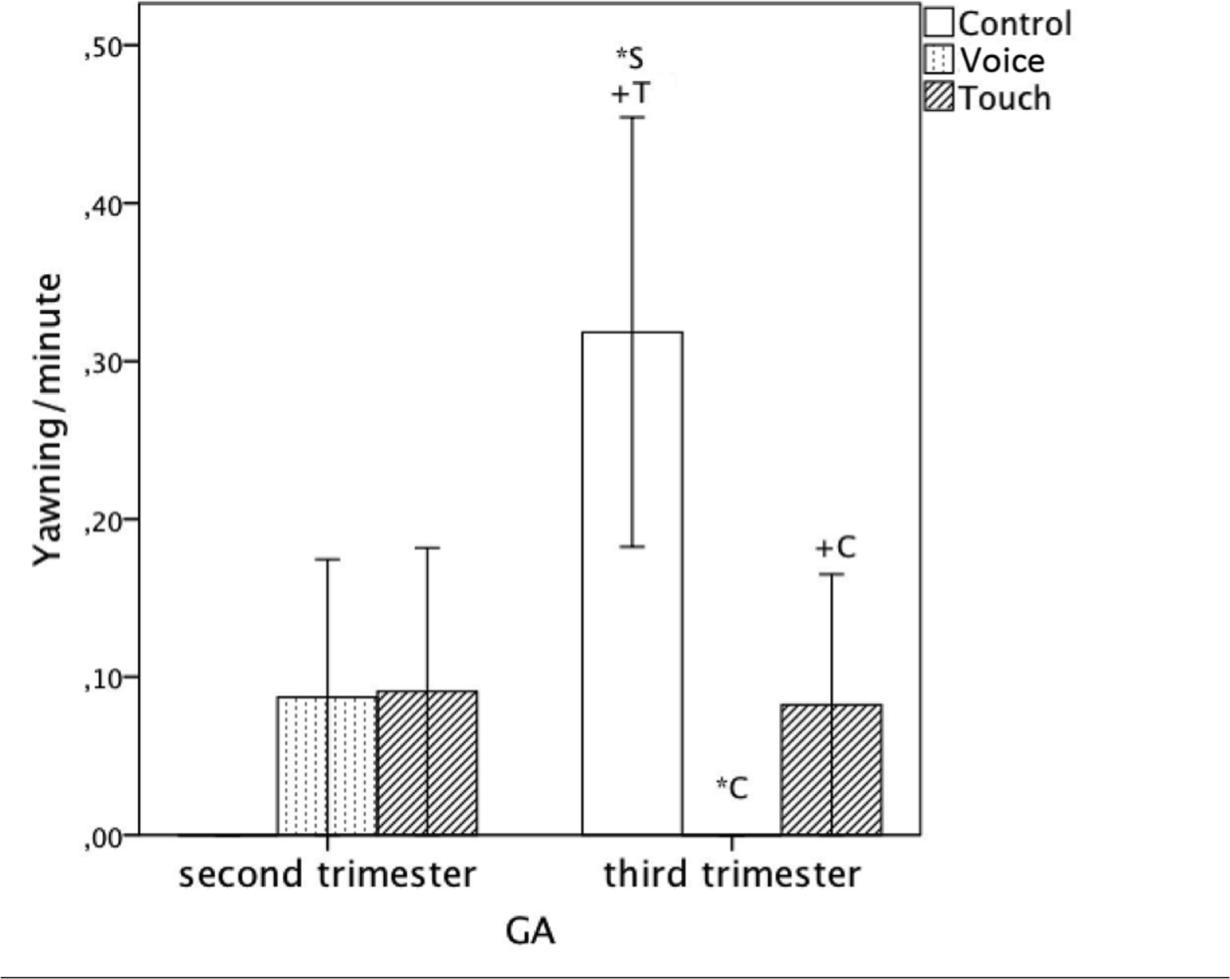
Average frequency of yawning per minute including standard errors for all three conditions (‘Control’, ‘Voice’ and ‘Touch’) between second and third trimester. +: p<.10 *: p<.05.

Older fetuses (third trimester) decreased yawning in both ‘Voice’ compared to ‘Control’ (‘Voice’: Mean = -1.388 E-17, *SE* = .06, ‘Control’: Mean = .32, *SE* = .10, *p* = .033) and ‘Touch’ (‘Touch’: Mean = .08, SE = .09, *p* = .12) while younger fetuses showed no statistically significant differences in yawning frequencies across the three conditions (see Fig 9, Table 3).

**Table 3:** Means, Standard Deviations and significance of pairwise comparisons for conditions and ‘GA’ on the frequency of yawning of the fetus.

	Second Trimester (<26 weeks)			Third Trimester (> = 26)		
Conditions	Control	Voice	Touch	Control	Voice	Touch
Mean	.00	.09	.09	.32	.-1.388E-17	.08
SE	.10	.06	.09	.10	.06	.09
Control		N.S.	N.S.		p = .033	p = .119
Voice	N.S.		N.S.	p = .033		N.S.
Touch	N.S.	N.S.		p = .119	N.S.	

## Discussion

While previous research has mainly focused on FHR responses in reaction to maternal voice the current study measured fetal behavioural responses to three conditions: to maternal touch of the abdomen, to maternal voice compared to a control condition.

Overall results suggest that maternal touch of the abdomen was a powerful stimulus, producing a range of fetal behavioural responses. Fetuses displayed more arm, head, and mouth movements when the mother touched her abdomen as compared to maternal voice in situ. The increase in their activity was also indicated indirectly by the decrease of arm crossing movements in older fetuses. The difference in the responses by older and younger fetuses to maternal touch may lend support to the early observation of [22] that older fetuses respond preferentially to touch compared to younger fetuses.

As younger fetuses were in the second trimester, the results of this study also indicate that fetuses respond to touch much earlier than previously described [21]: in the 21^st^-25^th^ week rather in the 26^th^ week of gestation.

Just like arm and hand movements of neonates are far from being random [29, 30], previous research [30] suggests that fetal hand and arm movements might also be directed and intentional [31, 32,33]. Although it is speculative to suggest, it might well be that the increases in arm movements in response to maternal touch are also directed responses towards the source of the stimulation [34].

The decrease in arm and head movements as a response to maternal voice supports the results of [14] using direct maternal voice to stimulate the fetus. The authors reported a decrease in FHR to maternal voice in situ as well as an increase in FHR to recorded voice. Although the current study did not use recorded voice, the behavioural quieting to maternal in situ voice corresponds to the physiological response of decreased FHR measured by [14] as well as to the orienting physiological response [26, 17].

Our study also reported an interesting behavioural change with maturation, from the 2^nd^ to the 3rd trimester. Regardless of the experimental condition, fetuses in the 3^rd^ trimester displayed more self-touch (hands touching the body) when compared to fetuses in 2^nd^ trimester. This observed increase in self-touch might be due to the increased tactile sensitivity of the skin as fetuses develop. As a consequence fetuses may seek out proprioceptive stimulation just as neonates were reported to do [35].

Fetuses in the 3^rd^ trimester also spend more time with crossed arms compared to fetuses in the 2^nd^ trimester. This behaviour most likely indicates that the fetus is resting, and if so, this finding is in support of previous findings [23], which reported an overall decrease of movements as fetuses mature. However as the fetus grows rapidly during the third trimester, the uterine environment becomes increasingly smaller limiting fetal motor behaviour. Therefore less movement and more touching the body was to be expected. It is also space saving to fold the arms in front of the body during rest. However, fetuses in 3^rd^ trimester cross their arms more often in response to maternal voice as compared to the touch condition. This behaviour is one of the behaviour activity responses to maternal touch, thus a decrease in arm-crossing behaviour might be a consequence of the increase of other, arm, head, mouth movements and indicates an increased activity of the fetus.

Older, third trimester fetuses yawned more during maternal voice stimulation compared to the control condition and also showed a tendency to yawn more during maternal touch. Fetuses in the 2^nd^ trimester, however, showed no differential change in their yawning. The observed increase of yawning in older fetuses stands in contrast to reports [24], who found a decrease rather than an increase in yawning frequencies from 28 weeks of pregnancy. Although the mechanisms and functions of fetal yawning are still unexplored most recent theories suggest its connection to activity dependent brain maturation of regulatory behaviours [25]. Overall increased regulatory (yawning), resting (arms crossed) and self-touch responses to external stimuli were observed among older fetuses. Such results could reflect the maturation process of the nervous system as the fetus develops.

In summary, the results from this study suggest that fetuses selectively respond to external stimulation earlier than previously reported, fetuses actively regulate their behaviours as a response to the external stimulation, and that fetal maturation affects the emergence of such differential responses to the environment.

Mothers, fathers and other family members talk and even sing to the fetus throughout pregnancy with communicative intent. Many report changes in the fetal behaviour as a response to such communication. And although we used the term ‘touch’, the condition however was not direct skin-to-skin contact but an indirect stimulation of the fetus via stroking the abdomen applying slight pressure. Similarly to talking to the fetus, most mothers and even fathers attempt to communicate with and regulate the behaviour of the fetus via stroking of the mother’s abdomen as a response to the kicking or positional movements of the fetus. Even the expecting mothers’ mood is affected by massaging the abdomen resulting in reduced depression [36].

As earlier research by Zoia et al [31, 33] showed, the kinematic patterns of the movements of fetuses reflect intentional actions, and advanced motor planning, therefore it is plausible to suggest that the observed fetal responses to the voice and touch in the present study may have a communicative intent.
